# Evaluation of aromatherapy with lavender oil on academic stress: A randomized placebo controlled clinical trial

**DOI:** 10.1016/j.conctc.2019.100346

**Published:** 2019-03-15

**Authors:** Rizwan Ahmad, Atta Abbas Naqvi, Hamdan Moayed Al-Bukhaytan, Ahmed Habib Al-Nasser, Ali Hassan Baqer Al-Ebrahim

**Affiliations:** a Natural Products and Alternative Medicines, College of Clinical Pharmacy, Imam Abdulrahman Bin Faisal University, Dammam, 31441, Saudi Arabia; b Department of Pharmacy Practice, College of Clinical Pharmacy, Imam Abdulrahman Bin Faisal University, Dammam, 31441, Saudi Arabia; c College of Clinical Pharmacy, Imam Abdulrahman Bin Faisal University, Dammam, 31441, Saudi Arabia

**Keywords:** Academic stress, Pharmacy students, Aromatherapy, Randomized controlled trial, Lavender, Exam stress, Heart rate, Blood pressure

## Abstract

**Introduction:**

Academic stress is prevalent among pharmacy students. Several factors such as hectic schedules, courses and exam load as well as lack of recreational time during semester have been reported as determinants of academic stress. Studies revealed; the use of aroma oils especially with relaxant properties may help ease stress.

**Methods:**

This study aimed to investigate the effect of lavender oil on academic stress during exams in pharmacy students. A randomized-single-blind placebo-controlled trial providing aromatherapy with lavender oil as an intervention was conducted in male pharmacy students. The outcomes assessed included stress, stool pattern, headache and vital signs that comprised of systolic, diastolic blood pressure (SBP and DBP) and heart rate (HR). The study was approved from concerned authority and registered in ClinicalTrials.gov (NCT#03460626).

**Results:**

The placebo and experimental group showed a significant (p < 0.01) difference in stress score (F = 244.865, p < 0.0001), headache VAS score (F = 8.187, p < 0.0001), SBP (F = 11.141, p < 0.0001), DBP (F = 3.873, p < 0.001) and HR (F = 8.537, p < 0.0001); at during-exam time-point as compared to control group. No significance was achieved; among three treatment groups in stool pattern (F = 2.143, p > 0.05) and, at post-exam time-point (p > 0.05).

**Conclusion:**

Aromatherapy with lavender oil did not have any effect on academic stress.

**Trial registration:**

The study was registered prospectively on ClinicalTrials.gov (NCT#03460626) on 19th February 2018.

## 1 Background

Stress affects cognitive function through disturbance in behavior, thinking and mood. The mood disturbance may pose mental, physical and emotional problems that affects the learning as well as thinking capabilities of individuals [1]. Work load i.e. preparing for exams along with acquisition of knowledge, skills and attitudes have been reported as more stressful to cope up with, during university life [2]. Many studies have reported the stress-suffering conditions of medical students, particularly during exam seasons [[3], [4], [5], [6], [7], [8]], however no significant difference was observed for gender regarding level of stress during exams [9]. Besides course load and exams, quizzes and busy schedule as well as academic competition among students for higher grades may aggravate stress [6,8]. This academic competition in students may sometimes be unavoidable [10]. Alternative treatment namely aromatherapy, hydrotherapy and homeopathy are employed widely to reduce stress now-a-days.

Aromatherapy; an alternative treatment system where oils extracted from natural sources such as flowers, petals and bark of plants, are used to improve the physical and psychological conditions of stressed individuals [11]. These naturally extracted aromatic essences are applied to balance cognitive functions and memory retention. The aromatic essences/oils used in aromatherapy including lavender oil, rosemary oil, jasmine oil, peppermint oil etc. are believed to stimulate cognitive function and thus used for the purpose of pain relieving and mood enhancement [12]. Lavender oil is possesses the inherent properties of being easily applied internally, for aromatherapy as well as for massage therapy in many clinical studies where they revealed a positive outcome. Lavender oil reduces stress and produce relaxation via limbic system, particularly the amygdala and hippocampus [13]. A comparative study in thirty six (36) volunteers showed a reduced stress level in group using lavender oil for aromatherapy as compared to placebo group [14]. A study conducted at Hong Kong University revealed the fact; that aromatherapy may be effectively utilized as a tool to alleviate pain, reduce depression and stress in adults [15]. A study among students of Iran, revealed a reduction in test-related anxiety with the use of *Polianthes tuberosa* essential oil [16]. Similar study in students of Florida Atlantic University proved the vital effects of aroma oils and recommended the use of rosemary and lavender oil during exams for reducing stress level [17]. In addition, lavender oil has been reported as stress reducer and to produce relaxation during exams [18]. Furthermore, the cream of lavender oil along with a foot-bath may be used in pregnant as well as non-pregnant females to reduce stress and anxiety [19,20].

Hence, lavender oil may be effectively used to reduce stress however; none of the studies have reported the use of lavender oil in pharmacy students particularly in stress during exams. Previous studies have highlighted that pharmacy students at Imam Abdulrahman Bin Faisal University, Dammam, Saudi Arabia suffer from severe stress especially during examinations [6,7]. Therefore, this study was designed to evaluate the effect of aromatherapy i.e. lavender oil in reducing academic stress among pharmacy students during exams.

## 2 Methods

A randomized-single-blind placebo controlled clinical trial providing aromatherapy with lavender oil as an intervention was conducted in pharmacy students enrolled at College of Clinical Pharmacy (male campus), Imam Abdulrahman Bin Faisal University (IAU), Dammam, Saudi Arabia. The duration of the study was two months and began on February 02, 2018 till April 05, 2018.

### 2.1 Target population, exclusion criteria and recruitment

The study included male pharmacy students who reported a satisfactory general health and well-being and were willing to participate. Students from other colleges and non-consenting students were not included [6,7]. Students diagnosed with central nervous system (CNS) disorders stress, anxiety and depression were not included. Students with chronic diseases, allergy to aromatherapy or lavender oil were also excluded. Recruitment was completed within a week's time through university student portal system.

### 2.2 Sampling strategy and sample size calculation

Random sampling was conducted to select students. The total number of male students enrolled in College of Clinical Pharmacy in 2017–2018 according to the University's portal, was 165. This figure was assumed as total population. Sample size was calculated by online calculator and was reported to be 116 [21].

### 2.3 Treatment groups, randomization

This study included 116 volunteers divided randomly into three groups as; Group-I (Control group: without any intervention/treatment) Group-II (treated with a placebo that was an odorless oil without any therapeutic effect i.e., almond oil) and Group-III (Treated group: administered with lavender oil). Simple randomization was carried out by student coordinator (third party) enrolling every odd numbered volunteer in placebo group and even numbered volunteer in intervention group. Every alternate odd and even numbered volunteer was enrolled in control group. Randomization sequence was generated using students' identity numbers from University's portal. The investigators were blinded to the assigned groups.

### 2.4 Aroma oil used in treatment groups

Lavender oil with a final concentration of 3% using almond oil (90%) as diluent was used in the experimental group. The oil after dilution and final preparation were transferred to proper clean vial in a fix amount (30 ml) and given to the volunteers. Same procedure was repeated for placebo group however, lavender oil used was not included in the preparation. No intervention was performed in the control group.

### 2.5 Mental orientation sessions

In the beginning an orientation lecture was conducted regarding stress management followed by a reinforcement lecture in mid semester.

### 2.6 Aroma sessions

The volunteers received an aroma session twice a day (thirty minutes each) for three weeks during final exams under the supervision of principal investigator and a trained nurse. The frequency of aroma session was considered based upon the availability of students during exam time. Furthermore, all the volunteers were trained regarding the use of aroma oil before the study, with the help of experts including the principal investigators and a nurse specialized in massage therapy, from King Fahad University Hospital (KFUH), Khobar, Saudi Arabia.

### 2.7 Outcomes and measures

#### 2.7.1 Perceived academic stress

A visual analog scale (VAS) was applied to document self-perceived stress, indicating various levels of stress from zero to five i.e. 0 = no perceived academic stress to 5 = severe perceived academic stress) [22]. Change from baseline perceived academic stress at day 7 (1st follow-up) and at day 14 (2nd follow-up) was documented.

#### 2.7.2 Stool pattern

Bristol Stool Chart was used to document stool pattern during examination [23]. Change from baseline stool pattern at day 7 (1st follow-up) and at day 14 (2nd follow-up) was documented. An improvement was expected in the outcomes specified in the methodology, i.e., blood pressure, perceived stress and bowel frequency. The former two outcomes were measurable through sphygmomanometer and Likert scale. However, there was no scale or objective method to measure student reported changes in stool consistency and frequency. Thus Bristol Stool Chart is a best-fit for this parameter as it could be easily given to students and they could select the picture that best related to their condition.

#### 2.7.3 Headache

A visual analog scale (VAS) was used to document the intensity of headache [24]. Change from baseline headache VAS score at day 7 (1st follow-up) and at day 14 (2nd follow-up) was documented.

#### 2.7.4 Systolic and diastolic blood pressure (SBP and DBP); heart rate (HR)

A mercury sphygmomanometer was used to note systolic blood pressure (SBP), diastolic blood pressure (DBP) and heart rate (HR) as described by Pickering and Stevens [25]. Change from baseline SBP, DBP and HR at day 7 (1st follow-up) and at day 14 (2nd follow-up) was documented.

#### 2.7.5 Statistical analysis/data analysis

**Fig. 1:**
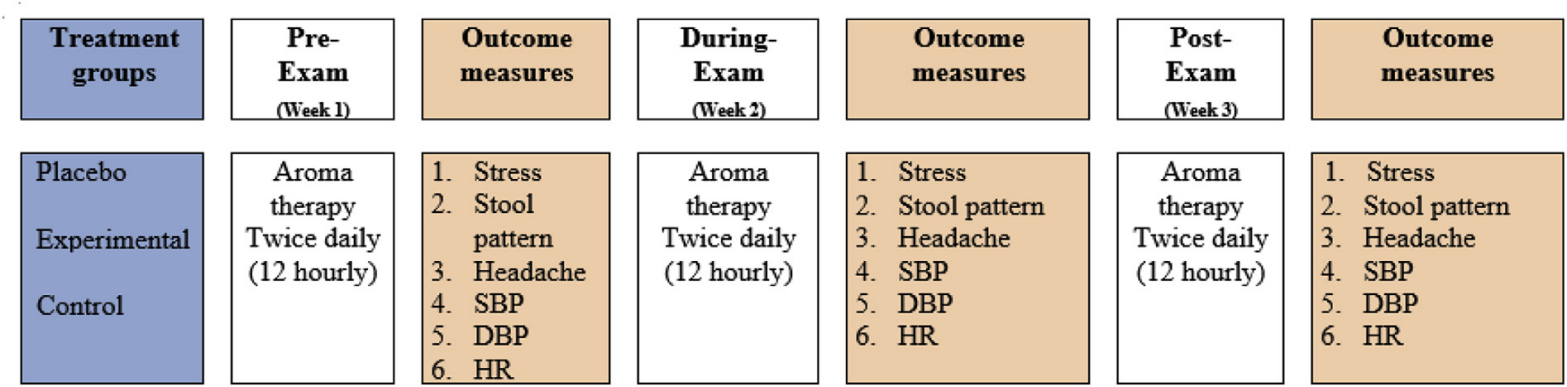
Study design.

The data was analyzed using multivariate analysis of variance (MANOVA) based repeated measures analysis or repeated measures MANOVA. For multivariate analysis the treatment group was considered as an independent variable (IV) and dependent variable (DV) included stress index, stool consistency, headache visual analogue scale (VAS), systolic blood pressure (SBP), diastolic blood pressure (DBP) and heart rate (HR). All DV are expressed in mean (X) and standard deviation (SD). The study design is presented in Fig. 1.

### 2.8 Ethics approval and consent to participate

The study was approved by Institutional Review Board, Imam Abdulrahman Bin Faisal University, Dammam, Saudi Arabia (ID # IRB-UGS-2018-05-060) and registered on ClinicalTrials.gov (NCT#03460626). Furthermore, a written consent was sorted from volunteers.

## 3 Results

A total of 116 students participated in the clinical trial. The treatment group consisted of three arms namely placebo group that enrolled 42 students, experimental group with 38 students and control group that had 36 students. No volunteer dropped out of the study.

### 3.1 Test for assumptions

Prior to analysis, several assumptions were tested. The dependent variables (DV) namely perceived academic stress, stool pattern; headache visual analogue scale (VAS), systolic blood pressure (SBP), diastolic blood pressure (DBP) and heart rate (HR) were checked for any outliers using mahalanobis distance. All DV had mahalanobis distance<16.27 (for 3 variables) which established that there were no outliers [26].

Secondly, the multivariate normality of data was analyzed using Shapiro-Wilk test of normality. All variables i.e., relating to stress, stool pattern, headache VAS and BP had p-value>0.05 that highlighted that the data for these variables was normally distributed. Hence, the null hypothesis is accepted for afore mentioned variables; the data had multivariate normality and that there were no outliers for all DV.

Second assumption was to test the hypothesis for multicollinearity. The authors assumed the hypothesis; that all DV had no multicollinearity. This was tested using Pearson's correlation. A correlation coefficient value less than 0.075 and p-value>0.05 indicated no multicollinearity [27]. The data established no multicollinearity among DV. The equality of covariance of DV across groups was analyzed using Box's test of equality of covariance matrices. A p-value>0.001 established that the covariance was equal across DV groups.

Wilk's Lambda (λ) was used to interpret the significant difference between IV and across linear time-points of DV. Homogeneity test for general characteristics between the groups was performed. Simple contrast k-matrix was employed to observed significant difference between two groups of IV at 3 linear time-points for a DV. Post-hoc analysis was conducted using Scheffe test since the treatment groups had dissimilar sample counts [28].

### 3.2 Mean change in perceived academic stress score at during-exam and post-exam times

The students indicated their perceived academic stress on a stress numeric rating scale consisting of scale of 0–5. A score of 0 indicated no perceived academic stress while score of 5 indicated severe stress. The mean change in perceived academic stress across three time-points was observed. The mean reduction in perceived academic stress from during-exam time to post-exam time-points was 0.28, 0.37 and 2.88 for placebo, experimental and control groups.

Multivariate analysis taking IV of treatment group with DV of stress index highlighted that there was a statistically significant difference (Wilk's λ = 0.808, F = 10.178, df = 6, p-value<0.0001) across three treatment groups on a linear time-point of DV. There was a statistically significant difference in perceived academic stress among treatment groups at during-exam time-point (F = 20.866, p-value<0.0001). However, there was no significant difference among the treatment groups at post-exam time-point (F = 2.581, p-value>0.05).

Contrast K-matrix revealed that there was a significant difference between placebo and control groups at during-exam time-point (p-value<0.001) however, it was not significant at post-exam time-point (p-value>0.05). Furthermore, there was statistically significant difference between experimental and control groups at during-exam (p-value<0.001) and post-exam time-points (p-value<0.05).

**Fig. 2:**
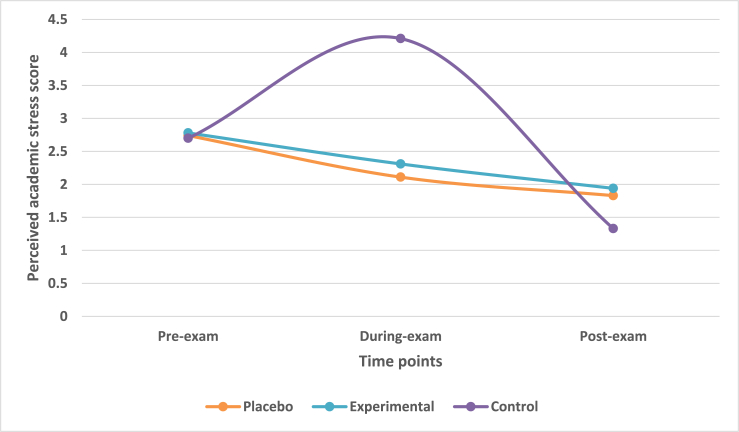
Mean change in stress scores in treatment groups across three time points.

Post-hoc Scheffe analysis revealed a non-significant difference (p-value>0.05) between placebo and experimental group at during exam and post-exam times. However, there was statistically significant difference (p-value<0.05) between control and placebo as well as between control and experimental groups at, during exam time-point. There was no significant difference (p-value>0.05) at post-exam time for the same (Fig. 2).

### 3.3 Mean change in stool pattern at during-exam and post-exam times

The students indicated their stool pattern on Bristol Stool Chart. There were 6 types of stools mentioned with a diagrammatic expression as well as brief description regarding shape, size, consistency and frequency. Stools from type 1 and 2 indicated constipation, stools from type 5 and 6 indicated diarrhea. Type 3 and 4 were considered normal stool pattern [23]. The mean change in stool pattern across three time-points was observed. The mean value for stool type remained in type 3 (i.e. 3.44, 3.83 and 3.78) for placebo and (3.54, 3.94 and 3.87) for experimental group. The mean value for stool type remained in type 4 at pre-exam (3.47) and during-exam (4.2) and in type 3 (3.6) at post-exam for control group.

Multivariate analysis taking IV of treatment group with DV of stool pattern highlighted that there was no statistically significant difference (Wilk's λ = 0.25, F = 2.143, df = 6, p-value>0.05) across three treatment groups on a linear time-point of DV. There was no significant difference among the treatment groups at during-exam (F = 0.573, p-value>0.05) and post-exam time-points (F = 0.3, p-value>0.05).

**Fig. 3:**
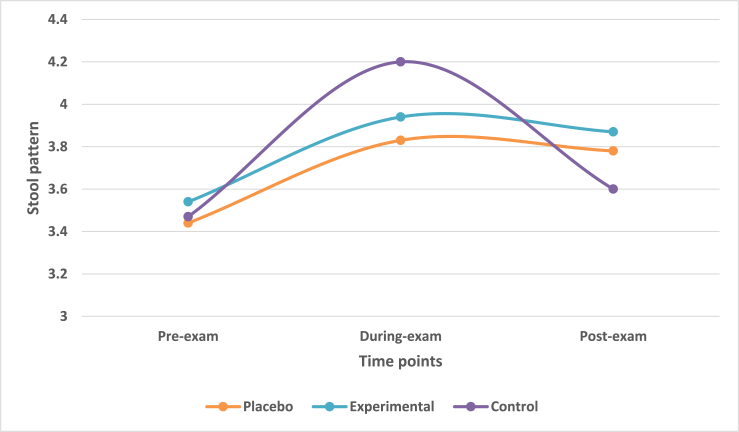
Mean change in stool pattern in treatment groups across three time points.

Contrast results highlighted that there was no significant difference between placebo and control groups at during-exam and post-exam time-points (p-value>0.05). No statistically significant difference between experimental and control groups was observed at pre-exam, during-exam time and post-exam time (p-values>0.05). Post-hoc Scheffe analysis highlighted that there was no statistically significant difference (p-value>0.05) between placebo and control group on any time-point. (Fig. 3).

### 3.4 Mean change in headache VAS score at during-exam and post-exam times

The students indicated their headache on a VAS consisting of scale of 0–10. A score of 0 indicated no headache while score of 10 indicated severe headache. The mean change in headache VAS score across three time-points was observed. The mean reduction in VAS score from during-exam to post-exam time-point in placebo and experimental group was 0.55 and 0.81 respectively. The mean headache VAS score in control group and decreased with a value of 5.54 at post-exam time.

Multivariate analysis taking IV of treatment group with DV of headache VAS score highlighted that there was a statistically significant difference (Wilk's λ = 0.706, F = 8.187, df = 6, p-value<0.0001) across three treatment groups on a linear time-point of DV. There was a statistically significant difference in scores among treatment groups at during-exam time (F = 28.989, p-value<0.0001). However, there was very weakly significant difference among the treatment groups at post-exam time (F = 0.184, p-value>0.05).

K-matrix revealed that there was a significant difference between placebo and control groups at during-exam time-point (p-value<0.001) however, it was not significant at post-exam time (p-value>0.05). Same was observed for difference between experimental and control groups.

**Fig. 4:**
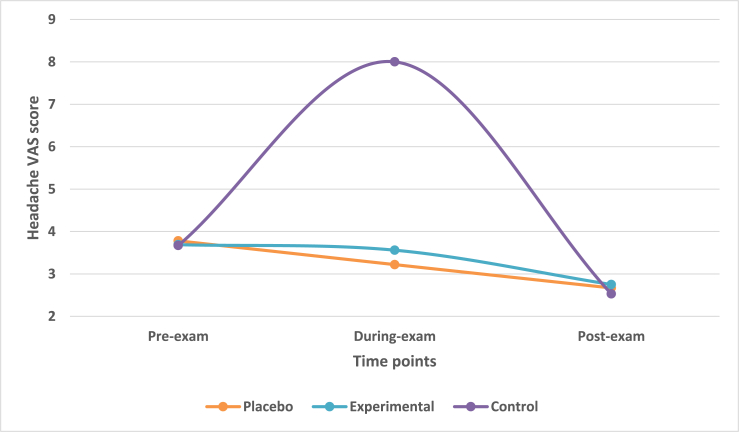
Mean change in headache VAS scores in treatment groups across three time points.

Post-hoc Scheffe analysis revealed a non-significant difference (p-value>0.05) between placebo and experimental groups at during exam and post-exam time-points. However, there was statistically significant difference (p-value<0.001) between control and placebo as well as between control and experimental groups at during-exam time-point There was no significant difference (p-value>0.05) at post-exam time for the same (Fig. 4).

### 3.5 Mean change in systolic blood pressure (SBP) at during-exam and post-exam times

Systolic blood pressure (SBP) was recorded using a mercury sphygmomanometer. The mean change in SBP across three time-points was observed. The mean reduction in SBP from during-exam to post-exam time-points, in placebo and experimental group was 1.83 and 5 respectively. The mean reduction in SBP from during-exam to post-exam in control group was 23.73.

Multivariate analysis taking IV of treatment group with DV of SBP highlighted that there was a statistically significant difference (Wilk's λ = 0.323, F = 11.141, df = 6, p-value<0.0001) across three treatment groups on a linear time-point of DV. There was a statistically significant difference in SBP among treatment groups at during-exam time (F = 26.380, p-value<0.0001). However, there was no significant difference among the treatment groups at post-exam time (F = 2.201, p-value>0.05).

Contrast analysis revealed that there was a significant difference groups at and during-exam time (p-value<0.001) however, it was not significant at post-exam time (p-value>0.05). This was applicable for difference between placebo and control groups as well as experimental and control groups.

**Fig. 5:**
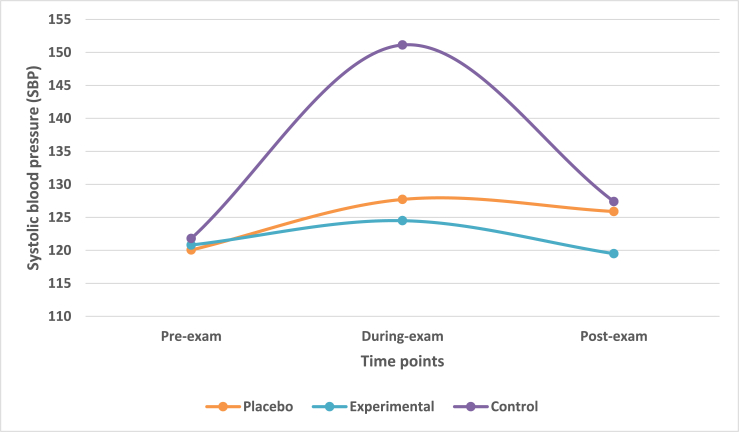
Mean change in SBP in treatment groups across three time points.

Post-hoc Scheffe analysis revealed a non-significant difference (p-value>0.05) between placebo and experimental group at during exam and post-exam times. However, there was statistically significant difference (p-value<0.0001) between control and placebo as well as between control and experimental groups at during exam time-point. There was no significant difference (p-value>0.05) at post-exam time for the same (Fig. 5).

### 3.6 Mean change in diastolic blood pressure (DBP) at during-exam and post-exam times

Diastolic blood pressure (DBP) was also recorded using a mercury sphygmomanometer. The mean change in DBP across three time-points was observed. The mean reduction in DBP from during-exam to post-exam time-points in placebo and experimental group was 1.11 and 1.38 respectively. The mean reduction in DBP from during-exam to post-exam in control group was 11.6.

Multivariate analysis taking IV of treatment group with DV of DBP highlighted that there was a statistically significant difference (Wilk's λ = 0.590, F = 4.427, df = 6, p-value<0.001) across three treatment groups on a linear time-point of DV. There was a statistically significant difference in DBP among treatment groups at during-exam time-point (F = 13.385, p-value<0.0001). However, there was no significant difference among the treatment groups at post-exam time-point (F = 1.935, p-value>0.05).

K-matrix revealed that there was a significant difference groups at during-exam time-point (p-value<0.001) however, it was not significant at post-exam time-point (p-value>0.05). This was applicable for difference between placebo and control groups as well as experimental and control groups.

**Fig. 6:**
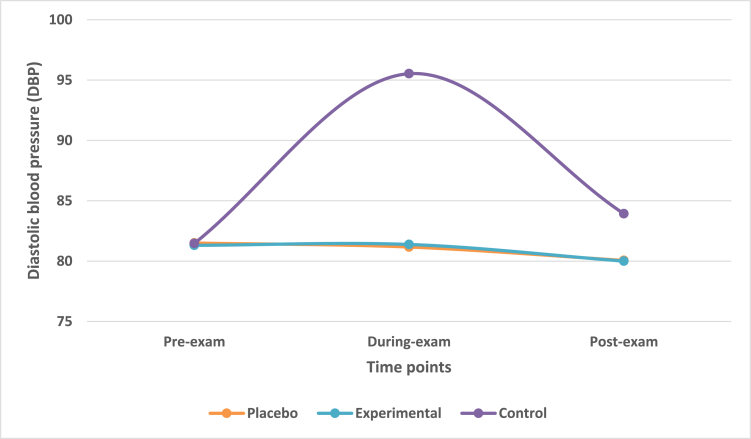
Mean change in DBP in treatment groups across three time points.

Post-hoc Scheffe analysis revealed a non-significant difference (p-value>0.05) between placebo and experimental group at during exam and post-exam time-points. However, there was statistically significant difference (p-value<0.0001) between control and placebo as well as between control and experimental groups (p-value<0.001) at during-exam time-point. There was no significant difference (p-value>0.05) at post-exam time-point for the same (Fig. 6).

### 3.7 Mean change in heart rate (HR) at during-exam and post-exam times

The mean change in heart rate (HR) across three time-points was observed. The mean reduction in HR from during-exam to post-exam in placebo and experimental group was 1.22 and 0.75 respectively. The mean reduction in HR was 33.07 bpm from during-exam to post-exam in control group.

Multivariate analysis taking IV of treatment group with DV of HR highlighted that there was a statistically significant difference (Wilk's λ = 0.290, F = 12.590, df = 6, p-value<0.0001) across three treatment groups on a linear time-point of DV. There was a statistically significant difference in HR among treatment groups at during-exam time-point (F = 27.143, p-value<0.0001). However, there was no significant difference among the treatment groups at post-exam time-point (F = 1.509, p-value>0.05).

Contrast analysis revealed that there was a significant difference groups at during-exam time-point (p-value<0.001) however, it was not significant at post-exam time (p-value>0.05). This was applicable for difference between placebo and control groups as well as experimental and control groups.

**Fig. 7:**
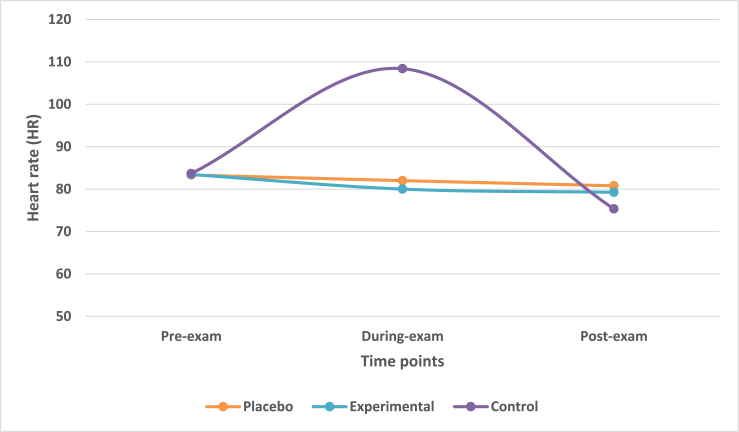
Mean change in heart rate (HR) in treatment groups across three time points.

Post-hoc Scheffe analysis revealed a non-significant difference (p-value>0.05) between placebo and experimental group at during exam and post-exam time-points. However, there was statistically significant difference (p-value<0.0001) between control and placebo as well as between control and experimental groups (p-value<0.001) at during exam time-point. There was no significant difference (p-value>0.05) at post-exam time for the same (Fig. 7). The mean differences in three time-points for three treatment groups is presented in Table 1 and outcome effect and significance in Table 2.

**Table 1:** Mean differences in outcomes for treatment groups.

Treatment groups	Perceived academic stress score Mean (Standard deviation)		
	Pre-exam	During exam	Post-exam
Placebo	2.74 (0.922)	2.11 (0.963)	1.83 (0.924)
Experimental	2.78 (0.885)	2.31 (0.873)	1.94 (0.854)
Control	2.70 (0.676)	4.21 (0.660)	1.33 (0.488)
	**Stool pattern** **Mean (Standard deviation)**		
Placebo	3.44 (0.705)	3.83 (1.2)	3.78 (0.943)
Experimental	3.54 (1.124)	3.94 (0.772)	3.87 (0.806)
Control	3.47 (0.884)	4.2 (0.941)	3.6 (0.507)
	**Headache VAS score** **Mean (Standard deviation)**		
Placebo	3.78 (2.016)	3.22 (2.238)	2.67 (2.326)
Experimental	3.69 (2.522)	3.56 (2.394)	2.75 (2.145)
Control	3.67 (1.033)	8.07 (0.884)	2.53 (0.915)
	**SBP** **Mean (Standard deviation)**		
Placebo	120.06 (12.986)	127.72 (12.043)	125.89 (11.499)
Experimental	120.81 (12.963)	124.5 (13.166)	119.5 (12.665)
Control	121.2 (6.930)	151.13 (6.812)	127.4 (9.179)
	**DBP** **Mean (Standard deviation)**		
Placebo	81.50 (9.721)	81.17 (8.319)	80.06 (6.743)
Experimental	81.31 (8.064)	81.38 (8.555)	80 (6.603)
Control	81.47 (10.378)	95.53 (9.884)	83.93 (5.725)
	**HR** **Mean (Standard deviation)**		
Placebo	83.33 (12.779)	82 (10.572)	80.78 (9.117)
Experimental	83.44 (12.215)	80 (11.261)	79.25 (9.154)
Control	83.67 (14.583)	108.4 (14.192)	75.33 (9.155)

**Table 2:** Outcome effect.

Effect	F	P-value
Δ in perceived academic stress	244.865	0.0001
Δ in stool pattern	2.143	0.056
Δ in headache VAS score	8.187	0.0001
Δ in SBP	11.141	0.0001
Δ in DBP	3.873	0.001
Δ in HR	8.537	0.0001

## 4 Discussion

The study was designed to evaluate the effect of aromatherapy on academic stress during exam times in undergraduate pharmacy students. The volunteers were randomly divided into three groups consisting of placebo, experimental and control group. All the volunteers were informed about this activity and ethical approval was obtained for the study. Furthermore, a written consent was sorted from volunteers and data was collected by either using validated tools or standard measures.

MANOVA with repeated measures technique was used to analyze the data [29]. Prior to conducting the analysis, the data was checked for various assumptions i.e. outliers, multivariate normality and multicollinearity. Based on assumption testing interpretation measures were selected. A considerable change in stress score was noted among treatment groups at pre- and during exam time points. There was no significant difference at post-exam for any treatment. The difference was between placebos and control as well as experimental and control however statistical significance failed to be achieved between placebos and experimental. This means, although stress was greatly reduced among treatment groups during exam however, the difference in stress reduction was almost same in placebo and experimental groups.

The reduction in stress level due to lavender oil, is in-line with previously reported studies where an important association have been established for aromatherapy in various stress conditions such as stress in nursing students [30], stress and anxiety due to hemodialysis [31], stress of nurse working in operating room [32], adolescent stress [33], stress due to workplace [34] as well as stress in college women due to dysmenorrhea [35]. However, when it comes to placebo vs experimental groups, a similar effect was observed in reducing the stress. The lack of difference between placebo and experimental group, in terms of superior effect, has been reported in few studies.

Anderson et al., in a study reported same effects for placebo and aromatherapy in post-operative nausea [36]. In addition, no significant difference for placebo and experimental groups was observed in treatment of distress in children's undergoing stem cell transplantation [37], treatment of agitation in Alzheimer's disease [38] visceral pain sensitivity between experimental and placebo groups [39] as well as a significant placebo-related hypoalgesia as observed by Charron et al., [40]. Many such studies are available where a significant effect from placebo group was observed as compared to experimental group.

Various popular beliefs are available to support the role of placebo in various clinical trials. Placebo may exert its role due to psychological and mental effectiveness and this theory is the most commonly believed one. The belief and hope of a person about treatment along with the suggestibility, positively affects the biochemical as well as the sensory experience of a subject towards a treatment [41]. The process-of-self-treatment is another factor contributing to dominant placebo effects. Any treatment system comprising of a care, attention, discussions, and affection to patient encourages confidence and hope which results in physical reactions and hence treatment [42]. In addition, the most widespread belief for placebo effectiveness is conditioning theory also called learning through association, “a process where a subject or patients learns from experience” [43]. It is best explained with the example of a patient taking aspirin on a regular basis for headache where he finds relief. If unknown to him, he is provided with the same tablet (same color, size and shape), he obtains the same relief [44].

In the current scenario the same factors of physical and psychological effects along with conditioning were assumed. Since the students were blind to the nature of oil i.e. whether lavender or placebo, however with the passage of time and through association they assumed the placebo to be a true lavender oil and thus reported a psychological effect regarding stress and other vital signs.

A significant difference was noted for change in headache VAS score among three treatment groups during exam. This difference was between control group and; placebo as well as experimental groups. However, there was no difference between placebo and experimental groups. It meant that placebo and lavender oil have similar effects in headache improvement. Lavender oil has been reported to be a safe and effective treatment in management of acute headaches [45] as well as tension type headaches in students [46]. The results from our study also support the effectiveness of lavender oil/aromatherapy in reducing pain intensity in stress related headaches. For placebo, effects like experimental groups are also reported [47] and the factors imparting such properties to a placebo-treatment have been discussed before.

The same effect was noted for reduction of systolic and diastolic blood pressure as well as heart rate. These results may be supported by the effect of lavender oil in reducing blood pressure through olfactory stimulation [48], reduction in blood pressure and heart rate through a decrease in autonomic arousal [49], a decrease of systolic and diastolic blood pressure along with a decrease in heart rate in individuals subjected to exercise [50], controlling the blood pressure and heart rate in patients undergoing coronary angiography through reduction in stress response [51] and a significant reduction of heart rate and blood pressure in patients going for open heart surgery [52].

There was no significant effect among the three groups at post exam time. This is logical as students will be relieved of their exam stress after completion of exams. Thus, any significance at this time point was not achieve.

From the results we could not establish any superiority of lavender oil over placebo in improving any of the outcomes i.e. stress, headache, stool, SBP, DBP and HR. The results support the hypothesis that complementary and alternative therapies are “no more effective than placebo” [53]. Though the mentioned reasons i.e. physiological and psychological effects of placebo, conditioning/learning through association and process of self-treatment, may contribute to lack of significant difference among placebo and experimental group however further studies with larger sample size are required to evaluate the difference between these two groups.

## 5 Conclusion

Lavender oil reduced student reported stress, headache VAS score and vital signs (SBP, DBP and HR) as like placebo, during-exam time. However, placebo along with above mentioned reduction in outcomes, improved stool pattern as well. Aromatherapy with Lavender oil is as effective as placebo in exerting its biological effect and no significance difference was found in decreasing the stress level and vital signs. Therefore, aromatherapy with lavender oil did not affect academic stress among pharmacy students.

## Declarations

### Ethics approval and consent to participate

The study was approved by Institutional Review Board of Imam Abdulrahman Bin Faisal University, Dammam, Saudi Arabia with ID # IRB-UGS-2018-05-060 and registered on ClinicalTrials.gov (NCT#03460626). Furthermore, a written consent was sorted from volunteers.

### Consent to publish

A written informed consent for publication was obtained.

### Availability of data and materials

The data set generated from the study is an intellectual property of Research Committee, College of Clinical Pharmacy, Imam Abdulrahman Bin Faisal University, Dammam, Saudi Arabia. It is not available publicly however, will be available from the corresponding author on genuine requests.

### Competing interests

None declared.

### Funding

No funding was sought and obtained for the study.

### Limitation of the study

The time management of students particularly during exam times was observed as major limitation. Similarly, the study included only male students. Non-inclusion of female participants was identified as a significant limitation towards generalizing the findings of our study. Further studies that include female participants are recommended in order to improve the validity of the results in this population.

### Author's contribution

RA conceived the idea with AAN, designed the study with HMA, AHN and AHB. RA wrote the introduction with HMA, AHN and AHB. HMA, AHN and AHB conducted literature review. RA and AAN designed the data collection tool and wrote the abstract and methodology. The data was collected and entered in SPSS by HMA, AHN and AHB. AAN conducted statistical tests and wrote the results section. RA and AAN wrote the abstract, discussion and conclusion with assistance from HMA, AHN and AHB. AAN edited the final draft of the manuscript. All authors read and approved the manuscript.
